# Building Evidence for Pre-school Policy Change in Bulgaria

**DOI:** 10.3389/fpubh.2021.594029

**Published:** 2021-04-16

**Authors:** Eugenia Volen, Joost de Laat

**Affiliations:** ^1^Early Learning and Care Program, Trust for Social Achievement, Sofia, Bulgaria; ^2^Utrecht Centre for Global Challenges and Utrecht School of Economics, University of Utrecht, Utrecht, Netherlands

**Keywords:** policy change, randomized controlled trial, advocacy, affordability of kindergarten, pre-school participation by disadvantaged groups, scientific evidence to policy

## Abstract

In September 2020, Bulgaria's National Assembly (Parliament) passed legal amendments aimed at increasing kindergarten participation for children aged 4–6 in Bulgaria, with poor children standing to benefit the most. For the first time, state budget funds were set aside to relieve parents of the costs of monthly attendance fees currently collected by full-day kindergartens. It builds on evidence generated from a large-scale randomized control trial (RCT) (2014–2018) across 236 poor communities, implemented by the Trust for Social Achievement (TSA) together with the World Bank and the Abdul Latif Jameel Poverty Action Lab. This paper describes how the RCT was used by TSA to advocate for removal of fees, and how much more evidence besides the RCT needed to be generated and support needed to be mobilized to influence policy action.

## Introduction

In September 2020, Bulgaria's National Assembly (Parliament) passed legal amendments aimed at increasing kindergarten participation for children aged 4–6 in Bulgaria through the removal or reduction of monthly attendance fees currently collected by kindergartens. Poor Bulgarian children, many though not all of whom are Roma ethnic minorities, stand to gain the most from this policy change.

With a young demographic in an otherwise aging general population, Bulgarians of Roma ethnic origin represent an estimated quarter of the country's new labor market entrants (1). Yet, low educational attainment levels among Roma contribute toward their exclusion from the formal labor market (2) and relegate many of them to a vicious cycle of intergenerational poverty. Fewer than 15% of Roma aged 18–24 graduate from high school, compared to a national average of 87% (3). Fewer than 1% of Roma are university graduates (4), and more than half work in low-skilled, low-paid jobs (5)^1^.

In 2012, the Trust for Social Achievement (TSA) was initiated, a grantmaking non-profit organization aiming to achieve systemic progress in Roma inclusion in several areas. Increasing participation in early education became a focus area from the beginning, drawing on multiple international longitudinal studies finding that quality early learning programs improve later life outcomes for disadvantaged children (6). While four out of five ethnic Bulgarian children attended pre-school in 2011, this was true for only two out of five Roma children (3).

To address the gap in kindergarten access TSA, in partnership with the World Bank and researchers^2^, designed a project called *Springboard for School Readiness* (SSR), consisting of several interventions implemented across 236 disadvantaged communities nationwide. TSA formulated the goal of the SSR as “*informing public policy on the most effective ways of increasing kindergarten participation by Roma children*.”

The SSR included a randomized control trial (RCT) to assess the impacts on kindergarten participation and child development. Before the SSR, conclusive evidence did not exist, and prejudices partially filled the void to claim that parental lack of interest explained the low participation rate of Roma children. The existence of financial barriers to attendance was at best privately acknowledged by decision makers, but not reflected in official policy.

This paper will explore how the SSR project evolved into a nationwide policy by 2020.

## Methodology

This study is based on an analysis of SSR related documents generated by TSA, the principal investigators of the RCT, and related documents produced by stakeholders such as the Ministry of Education and the Parliament. It is also informed by many discussions over an 8-year period with TSA staff, government representatives at national and local levels, and representatives of NGOs that participated in the implementation of the SSR. One of the authors, EV, has been with TSA since 2012, where she was involved in the SSR design and responsible for its implementation, outreach and advocacy efforts. The other author, JdL, has been one of the principal investigators on the RCT, also since the beginning. He was a Senior Economist and cluster lead for the World Bank's work on Roma inclusion until 2013 when the SSR was conceived. He is currently a professor of economics at Utrecht University.

## Policy Context

Prior to the SSR, legal amendments from 2010 provided for 2 years of compulsory pre-school education to encompass all 5 and 6-year-olds. In Bulgaria, kindergartens enroll children aged 3 and older, offer full-day services and collect attendance fees from parents on behalf of municipalities to cover, for e.g., daily meals and building maintenance. Schools only offer half-day pre-school education, where it is free of charge but without meals. Outside of big cities, not all settlements have both a kindergarten and school on their territory. At the time the SSR was launched in September 2014, participating in some form of pre-school program was thus *de jure* compulsory and free of charge for the 5 and 6-year-old children, but not necessarily *de facto*.

The Preschool and School Education Act (2015) (Education Act) governs the ability of municipalities to collect fees from parents. Municipalities receive funding from the state to cover teaching costs (salaries, books, education supplies), while maintenance, repairs and transportation are municipal responsibilities. Broadly speaking, the Ministry of Education and Sciences (Ministry of Education) proposes the type and amount of per-student and per-institution costs it will cover for municipalities; a suggestion that the Council of Ministers approves, and Parliament votes on through the annual state budget.

## SSR Project

### Preparation for the SSR Project 2012–2014

In 2012, the World Bank connected with the newly established Trust for Social Achievement to discuss collaboration on a RCT to assess barriers faced by poor and disadvantaged families, Roma minority children in particular, to participate in kindergarten.

At the time, the World Bank's Social Inclusion Project (SIP) 2009–2016 (7) provided EUR 40 million in loan financing to the Government of Bulgaria with the goal of promoting kindergarten participation among disadvantaged children (including children with disabilities and special needs). A significant portion of expenditures were directed toward infrastructure investments such as building new kindergartens or rehabilitating existing ones, and purchase of specialized equipment (e.g., transportation).

Additionally, the World Bank had been pursuing an active analytical program in support of Roma inclusion, including a widely referenced 2010 study on the economic benefits (1), a collaboration in 2011 with the European Commission and UNDP to conduct a large scale survey among ethnic Roma households across Eastern Europe (3), and a study focusing specifically on pre-school access among Roma across Eastern Europe (8).

These studies provided the economic rationale, documented the very large gap in pre-school participation between Roma and non-Roma, and pointed to self-reported barriers faced by parents. These studies also called for the use of impact evaluations to unpack, in scientifically robust ways using RCTs, the importance of specific barriers.

Together, TSA, the World Bank, and the principal investigators for the RCT, designed the SSR project to test three reasons why poor and disadvantaged parents in Bulgaria, Roma in particular, may not send their children to kindergartens to the same extent as other Bulgarian parents:

1. Parents may lack awareness about kindergartens, how to enroll, and the benefits for child development;
2. Parents may not be able to afford the fees that kindergartens collect;
3. Parents may be reluctant to enroll their children for other reasons.

To assess which one(s) of these is the most important barrier, TSA designed corresponding interventions:

1. A community campaign informing parents about the benefits of kindergartens;
2. Removal of the kindergarten attendance fees charged to parents;
3. Providing financial incentives to parents, conditional on children attending kindergarten regularly, in the form of food vouchers.

In 2012, with support from the World Bank's Bulgaria country office, the team requested, and received, a letter from the Bulgarian Minister of Education in support of the project and the accompanying RCT. TSA secured financing for the intervention from the America for Bulgaria Foundation and the principal investigators successfully applied for data collection funding to the World Bank's Strategic Impact Evaluation Fund (SIEF).

A pilot project was launched in four communities in 2013. In parallel, 236 communities throughout Bulgaria were identified, which were poor and had large shares of minority populations, and at the same time had a full-day kindergarten nearby with sufficient open places to accept new enrollments.

### SSR Project Implementation and Evaluation Year 1: 2014–2015

The SSR project began in earnest in Spring 2014. First, in April 2014, a community listing survey collected basic information on the 236 communities and their kindergartens. This was followed by a baseline survey in the same communities in April-May 2014. In each community, 25 children aged 3–6 were randomly selected, with 5,772 beneficiary children selected altogether by The Open Society Institute—Sofia (OSIS), which was contracted by the World Bank to collect the data under the guidance of the principal investigators.

Among the selected children, 75% were either Roma or Turkish, 62% did not speak Bulgarian at home, and most came from impoverished families. The average household monthly income, including government transfers, was 41% of the national average. Education levels among parents were very low: only 57% had completed primary school (9).

For the implementation of the SSR interventions, TSA selected through a competitive process 23 regional NGOs with a history of working with the local Roma communities and focusing on early education challenges. Each NGO was responsible for a distinct geographic region that included communities from all intervention groups. TSA issued grants to the NGOs and focused its own efforts on training them and the kindergarten representatives, reporting and stakeholder communications, and managing resource needs for the project as a whole (such as procuring food vouchers on a monthly basis). The SSR project was formally launched in June 2014, when TSA held a public lottery in Sofia in front of project stakeholders. The lottery randomly assigned the 236 communities to the different intervention groups, including a control group.

Over the course of the first year, OSIS carried out random spot checks to measure attendance and collect enrollment data from the kindergartens. In April–May 2015, it conducted a follow-up survey of parents to collect information on kindergarten participation, parental engagement with children at home (e.g., play), parental aspirations for their children, parental employment, etc. With support of Save the Children, OSIS also conducted a direct assessment of children to measure child development (motor skills, language and early literacy, math and problem solving, and socio-emotional growth) using the International Development and Early Learning Assessment (IDELA). This instrument was developed in 2011 by Save the Children and widely applied throughout the world in low- and middle-income countries (10).

The researchers found that the free access intervention is the most cost-efficient strategy to encourage participation of disadvantaged children in kindergarten. Removing fees increased kindergarten registration (enrollment) by 19% (13.4% points), reducing almost by half the proportion of children not enrolled. Free access also increased daily attendance by about 20%. The additional financial stimuli (food vouchers), which are an expensive component to administer (18–51% costlier than just covering kindergarten-related costs), did not yield incremental impacts on tracked outcomes. Information sessions were also effective at raising enrollment but did not affect daily attendance. When analyzing the impacts on child development, the researchers found that an increase in kindergarten participation was associated with some skill improvements for ethnic Bulgarian children, but slightly lower emergent numeracy and socio-emotional skills for minority children (9).

For the first time in Bulgaria, the SSR project had produced definitive proof about the lack of affordability of kindergarten services. However, the child development outcomes proved challenging to interpret. How can one argue to enroll disadvantaged children in kindergarten when it does not appear to boost assessed outcomes, at least not in the short run? Why isn't kindergarten helping all children develop equally? After all, Bulgaria's public kindergarten system has existed for over 130 years; it has broad geographic coverage and a body of professionally trained teachers: on average, SSR teachers had 20 years of experience, 43% had a masters' degree, and 31%—a bachelors' degree (11). These results came as a surprise and required careful follow-up analysis, preventing a quick release of the impact evaluation results and corresponding launch of TSA's advocacy strategy.

Some stakeholders wondered if the IDELA test focuses on academic knowledge such as reading and writing, which does not correspond well to the skills and knowledge nurtured through the curriculum in Bulgarian kindergartens, based around activities such as play, drawing and storytelling. However, the research team considered this unlikely as IDELA is a comprehensive test that also measures sub-skills such as listening comprehension and expressive vocabulary alongside print awareness and emergent writing (10).

A plausible explanation was that perhaps the quality of interactions between teachers and children, and teachers and parents was affected by a shortage of motivation, knowledge and skills to work with children living in poverty, as well as conscious and unconscious biases toward the Roma. Feedback collected from project teachers during two focus group discussions held in September 2016 and observations made by the TSA and NGO teams during the more than 1,600 SSR monitoring visits to kindergartens suggested that some teachers struggled to cope with the increasing proportion of disadvantaged minority children, while others exhibited unconscious biases as represented through statements such as “that's about as much as I can expect from my group of kids.” Other studies of kindergarten teacher sentiments have also pointed to the existence of racial biases and prejudices (12).

Moreover, perhaps one academic year of kindergarten exposure was simply too short a period to expect significant progress with children who mostly do not speak Bulgarian at home. Studies suggest that two or more years of kindergarten participation make a meaningful difference in fourth and seventh grade academic test scores of children whose mother tongue is not Bulgarian (13, 14).

### SSR Project Implementation and Evaluation Continuation: 2015–2018

**Figure 1:**
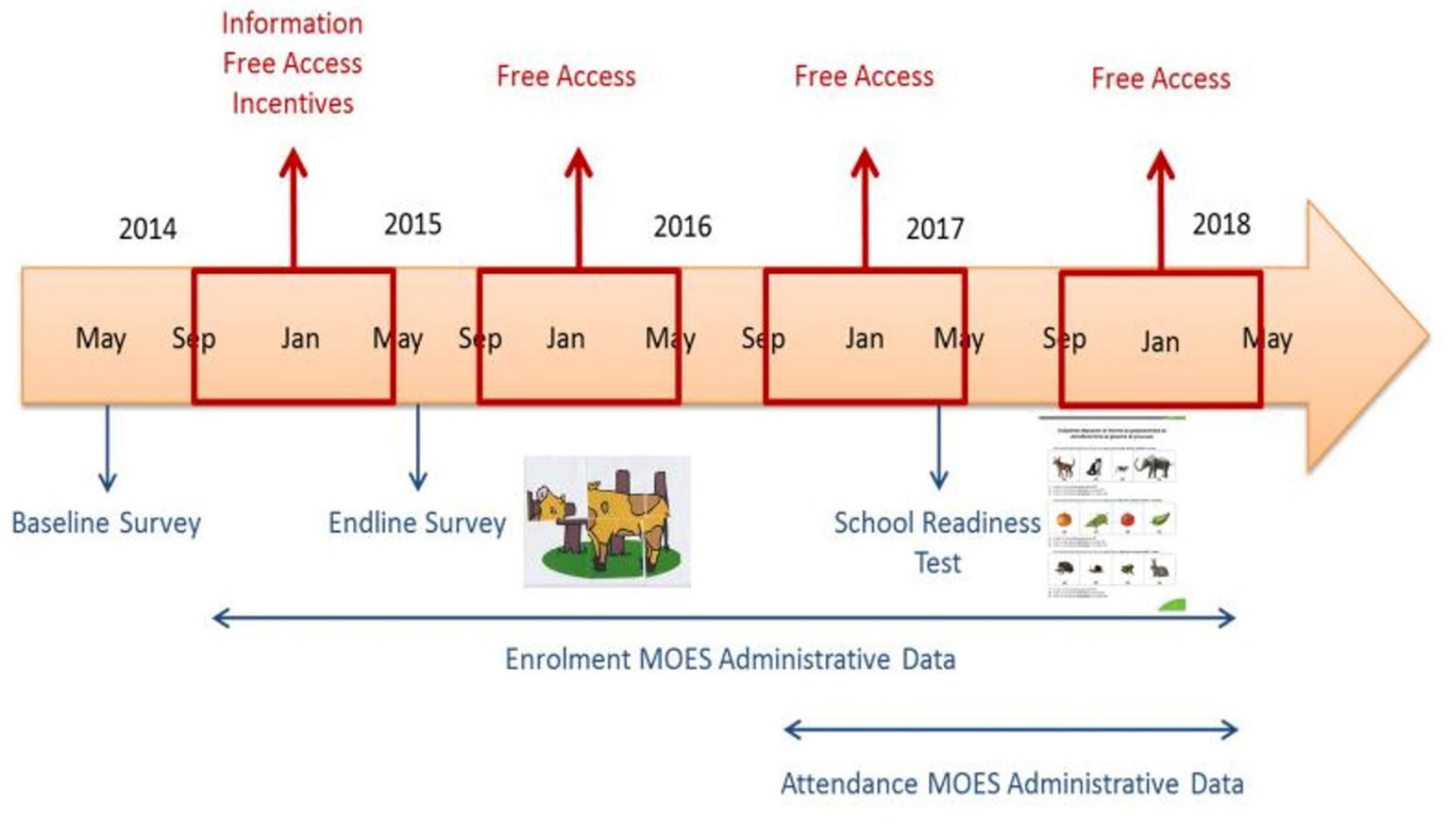
Combined timeline of Springboard to School Readiness 2014–2018, including the main interventions and data collection instruments applied. Adapted from: Huillery and de Laat (11). Supporting Disadvantaged Children to Enter Kindergarten: Second (Medium-Term) Follow-Up Study. Washington, DC: World Bank.

Motivated in part by these initial results, the project and evaluation were extended with continued financial support from the America for Bulgaria Foundation. The combined timeline of the 2014–2018 initiative, including the main interventions and data collection instruments applied, is illustrated in Figure 1 below.

A number of changes were implemented in the project. First, in the three academic years that followed, free access to kindergarten was continued in communities which were offered free access in 2014–2015. The financial incentives, however, were stopped. The information campaign also stopped at the end of the first year, as parents had already received the information. Further, TSA decided not to continue its collaboration with 5 of the original 23 NGOs responsible for implementing the project at the community level. Free access in the corresponding communities was discontinued resulting in 70% of all the original financial intervention communities continuing to receive free access during the 3 years that followed. This did not affect the validity of the RCT because the experimental design was stratified from the beginning by NGO.

In Spring 2017, a follow-on child development assessment was administered. At that time, the third and largest cohort of project children was about to graduate from kindergarten and transition to primary school. The hypothesis formed earlier—that it takes a few years of regular attendance to improve development outcomes for the most disadvantaged children—could now be confirmed. The test consisted of 40 questions and measured emergent literacy and numeracy of the child. The instrument was the Bulgarian “Standardized School Readiness Diagnostic Test,” published by the “National Test Center” association. Using a Bulgarian test would avoid criticism (such as the one encountered regarding the IDELA tool) that the instrument does not correspond to the Bulgarian pre-school curriculum.

The Ministry of Education also became more involved over this period. During 2014–2015, the Ministry formally supported the efforts and was the primary beneficiary of the research. With the follow-on study it provided administrative data on enrollment, attendance and teacher characteristics, and supported TSA to locate, train and instruct the 44 enumerators who administered the test.

The follow-on report, published by the World Bank in 2019, found that the negative 1-year impacts on child development had been reversed. By 2017, these same children scored significantly better than in the control communities. This demonstrated the importance of multiple years of kindergarten, especially for the most at-risk children. Still, some crowding out was observed among minority children who had already been enrolled at baseline and demonstrated lower school readiness results than the control after 3 years. Majority children involved in the study were unaffected by the provision of additional years of free access (11).

## Advocacy

### Preparing Advocacy for Policy Adoption

During the first phase of the SSR, success meant producing high quality evidence. In 2017, TSA shifted its goals from simply informing policy to actually *attracting support*, from awareness-raising to *convincing and bringing about incremental change*. TSA's efforts evolved to be guided by the number and types of people reached with the findings (as an output), how many of them were convinced, and how many acted on the information (as outcomes). In its advocacy, TSA made use of the “Advocacy Toolkit” developed by UNICEF (2010), which provides resources for an advocacy strategy, including eight foundational areas to strengthen internal advocacy capacity. The tool was used as a broad guide and sense-check of direction (15).

TSA established baseline levels for outcome indicators and informed its advocacy strategy by (1) counting the number of municipalities offering free or low-cost kindergarten, (2) commissioning a nationally representative survey on attitudes toward removal of kindergarten fees, and (3) assessing the capacity for advocacy of the SSR NGOs.

TSA obtained from the Ministry of Education data on fees charged by each municipality in Bulgaria and updated it annually. In early 2017, 18 out of 265 Bulgarian municipalities offered free or low-cost kindergarten. TSA initially set a goal for incremental change in 7 more municipalities over the course of the next 3 years.

The nationally representative survey established a baseline of support and guided TSA's strategy on which parents to advocate to and how. The survey revealed a significant disparity in opinions between the capital, on the one hand, and villages and small towns on the other. Many parents in the capital Sofia, faced with a chronic shortage of places in municipal kindergartens and forced to seek much more expensive private options, expressed concerns about exacerbating shortages and showed a strong preference to keep fees in place. Parents in villages and small towns overwhelmingly wanted kindergarten fees to be removed and saw the increased enrollment as a benefit. This result guided TSA's decision to advocate for flexible national policy solutions. Further, it informed a decision to focus local advocacy efforts on smaller municipalities (where the expressed need and level of public support was greater), while applying an awareness-raising strategy in the capital. Mirroring opinions of parents, Sofia city officials were not receptive to the idea of removing attendance fees. Achieving policy change in the capital was deemed too high a bar at that time, perhaps only possible through a national policy. Still, the potential for Sofia's priorities—especially parental opposition—to drive national initiatives could not be overlooked. Thus, to build a certain level of compassion and willingness to compromise, TSA considered it necessary to at least inform Sofia parents, municipal experts and NGOS about what needs outside of the capital look like.

Over the years, the NGOs involved in the SSR formed into a focused network. Starting in 2014, TSA organized regular experience exchanges at which representatives could discuss their work in the project. These conversations gradually evolved from discussing implementation issues to advocacy issues. A survey on institutional capacity for advocacy among these SSR network members further helped strengthen efforts by informing the design of training programs such as speaking to the media and writing success stories.

### Advocacy at Municipal Level

Initially, municipal officials seemed largely unconvinced of the RCT findings. For example, by demanding a breakdown just for their region, which was not possible given the RCT sample size. To target municipal advocacy, in 2017, TSA alongside OSIS and 16 of the original 23 NGOs, identified municipalities with favorable conditions for a policy revision using a matrix of 28 indicators in five areas: needs of the population, size effects of a possible policy to remove fees, level of perceived institutional support, level of perceived public support and perceived local strength of NGOs. Assessments were based on statistical data, key informant interviews, municipal reports and subjective experiences. The project team selected six municipalities for further research and advocacy. The assessments allowed the project team to raise awareness about SSR results in 30 municipalities.

At each of the six municipalities, the NGO partners identified and approached key local stakeholders; interviewed teachers, principals and parents to assess their attitudes and raise awareness; established a civil society expert task force and a parent volunteer group; and prepared an advocacy action plan together with these two support structures. The results were shared with municipal authorities, with presentations always combining the SSR impact evaluation, the national survey and the local qualitative research.

This proved to be a particularly successful approach as three of the six municipalities removed kindergarten fees or abandoned plans to increase them (Antonovo, Kuklen, Shumen). Another two (Pavlikeni, Peshtera) had just expressed willingness to consider such a policy when the national policy for kindergarten fee removal was announced in late 2019. TSA later replicated the model in one more municipality—Lukovit—where the local analysis revealed a lack of available places in kindergartens.

In addition to these concentrated efforts, during Spring 2019 the SSR NGOs carried out a nationwide campaign to present the SSR results and additional evidence to a further 20 municipalities, 23 kindergartens and 16 communities, reaching a total of 881 people. By 2019, the overall number of municipalities in Bulgaria that took steps to remove kindergarten fees had grown to 40 (15% of all municipalities in the country). TSA used the experiences from the municipal level, including the growing number of municipalities removing fees, to feed into its national level advocacy (and vice versa).

### Advocacy at National Level

Together with OSIS and the SSR network, TSA prepared a series of analytical reports on the international, national and local sources of funding for kindergartens, on municipal good practices with respect to removing fees, and on the potential cost of scaling a fee removal policy nationally. These reports helped answer questions such as “how much will fee removal cost under different scenarios?,” “where can the money from?,” and “how can it be implemented at scale in practice?” The cost analyses drew on data collected during the SSR project, among other sources. The project team also performed a stakeholder mapping exercise that identified concerned parties; researched their relative power, interest in and position on the issue; and developed targeted messages for each category. This activity allowed TSA to estimate the extent of initial institutional support for fee removal and guided next steps in differentiating advocacy tactics.

Through the mapping, TSA were able to identify allies and gain support from influencers and messengers ranging from musicians to politicians. It provided a starting point for initiating a series of meetings, roundtables, and conferences. For example, it connected TSA to the Ombudsman of the Republic of Bulgaria, who became a champion for the idea. She launched her own nationwide campaign in 2018 “Without fees in the kindergarten” (16). Efforts by allies such as these provided considerable national and local media coverage, reaching an estimated audience of over 60,000 people. Altogether, during 2017–2019 TSA presented the findings to over 600 stakeholders at 10 key events.

## Policy Adoption and Implementation

Statistical data suggests that the 2010 policy of 2 years of mandatory pre-school raised enrollment rates only temporarily as the rate had been steadily declining since 2014, stabilizing at 78% in 2018 (17), short of the EU-wide “Barcelona target” of 90% enrollment rate in early education by 2020 (18).

By early 2019, TSA and its SSR partners had been bringing up the issue of kindergarten affordability in person and at public events for so long now that the Minister and other education officials began pre-emptively discussing it. TSA had developed solid counter arguments on how such policies could benefit all stakeholders. The topic of removing kindergarten fees began making its way to public discourse even when neither TSA nor their direct partners were present.

In Spring 2019 the Ministry of Education negotiated with the European Commission the design of a new EU funded program that sets aside BGN 8.8 million (EUR 4.4 million) toward covering kindergarten attendance fees for children from disadvantaged communities (19). This national program was a milestone toward an actual national policy. That Fall, TSA and partners were invited by the Minister of Education to share the cumulative evidence and discuss how to address the complexity of local challenges. Several weeks later, the Ministry publicized its intention to propose an amendment to the Education Act compelling the state to set aside annual funds to cover kindergarten fees, citing some of the very arguments and ideas that TSA and partners had been promoting. To complement the fee removal, the Ministry also announced the launch of a parallel initiative worth BGN 70 million (EUR 35 million) aimed at expanding kindergarten places in locations such as Sofia and Lukovit. As a third and final measure to increase enrollment, the mandatory age for pre-school education would be lowered from five to four, giving municipalities up to 3 years to comply.

The final policy adopted by Parliament in September 2020 requires the state to provide funding to municipalities specifically earmarked for kindergarten fee removal for pre-school aged children (20). Empowering local authorities to decide which categories of children should be covered, the amendment mandates that at least half of the funds should be used toward the full removal of fees. The Ministry expects that over the next few years at least half of municipalities currently charging fees will leverage this central government funding to remove fees for all children of mandatory pre-school age. Still, the flexibility of the policy is likely to result in a diversity of local approaches and an extended implementation timeline. It was likely a necessary compromise to accommodate the very different needs of Sofia and the rest of the country. The stated policy aim is to gradually cover at least 90,000 children annually.

## Discussion

In 2012, TSA, the World Bank, and its research partners planned a RCT to inform national policy to improve kindergarten access. In 2020, Bulgaria adopted a new national policy to remove kindergarten fees for disadvantaged children.

Petticrew and Roberts (21) argue that a hierarchical approach to quality of evidence is unhelpful because “effective implementation of an intervention ideally requires both [qualitative and quantitative] sorts of information.” Indeed, this case study shows that it took a great amount of evidence (and much more) to help bring about systemic change over the course of 8 years. Out of this cumulative body of proof, only the RCT generated information that can be considered “Level I” or highest quality in the “hierarchies of evidence” (22, 23) widely applied in evidence-based practice across a number of fields.

Certainly, the RCT was very important, especially at the national level. The cumulative findings were relatively straightforward to communicate and provided an opportunity for advocacy. The RCT design lent credibility and boosted confidence among audiences. However, the RCT did not answer all questions relevant to systemic policy change, whether at the national or at the local level. In short, important, but insufficient. TSA and its partners collected additional evidence, both quantitative and qualitative, to assess the fidelity of the intervention; to estimate the cost of a national scale-up; to analyze parental attitudes toward policy change; and to better understand the extent of and approaches toward local policy take-up. Not only was this data used extensively for advocacy activities; it also helped form a baseline to measure the success of those very activities.

Furthermore, it also mattered *who* was presenting the evidence to audiences. Success required a sustained effort by a broad coalition of collaborators providing consistent messaging. Seven years were required to both broaden the coalition of stakeholders and to deepen their support. Aside from TSA and the World Bank, several key collaborators were involved from the beginning: a focused network of NGOs and the Ministry of Education. But it took time before they became powerful advocacy allies.

While the NGOs were initially selected to implement the SSR interventions in local communities, over time they began to carry out complementary analyses. Regular convenings by TSA provided an opportunity to further connect, learn, and become a coordinated network advocating for policy change. The Ministry of Education's commitment also grew as the years progressed, leading to the point where the Ministry became the owner of the issue, designing a policy, consulting it with interested parties and pushing it through Parliament for approval. The stakeholder mapping identified additional actors, who in turn became vocal supporters, such as the national Ombudsman and several political parties in Parliament.

Finally, while the RCT provided the scale of evidence needed, the average treatment effect measured through the RCT did not capture the variability in treatment in all circumstances from one locality to the other. Indeed, the policy decision making process is influenced by a number of factors beyond proof of policy effectiveness, from municipal budgetary constraints of the smaller municipalities or lack of available places in Sofia kindergartens, to the perceived societal acceptability of providing services for free without a corresponding obligation. Such considerations can significantly shape the ultimate policy adopted.

The shared goal of all stakeholders was to increase participation in kindergarten. The policy recommended by the SSR impact evaluation was to remove kindergarten fees for children living in poverty. The Ministry of Education augmented this proposal by adding funds for infrastructure development to address challenges in ensuring available places and by increasing to three the number of required years of pre-school education. The policy actually passed by Parliament transferred to municipalities the decision authority on whom to remove kindergarten fees for but provided sufficient restrictions and stimuli to guide a majority of municipal authorities toward covering all children. Moreover, the final policy also dedicated a sizeable annual budget toward reimbursing childcare costs of parents who are forced into private kindergartens owing to a deficiency of free places in municipal-run kindergartens. These last-minute changes were a direct result of pressure from informal parental associations in Sofia to provide immediate relief for the critical lack of open places in the capital's kindergartens.

In hindsight, by 2019 this body of evidence and the advocacy around it had created a tipping point in support of the policy change. Something which seemed so far out of reach in 2012, rather suddenly and quietly was passed into law by Parliament in 2020.

## Data Availability Statement

The original contributions presented in the study are included in the article/supplementary material, further inquiries can be directed to the corresponding author/s.

## Ethics Statement

Ethical review and approval was not required for the study on human participants in accordance with the local legislation and institutional requirements. Written informed consent for participation was not required for this study in accordance with the national legislation and the institutional requirements.

## Author Contributions

EV was involved in the project design and was responsible for its implementation, outreach and advocacy efforts. JL has been one of the principal investigators on the RCT since the beginning of the project. Both authors have been collaborating since 2013 and together planned the content of, wrote sections reflecting each side's perspective and experiences, and edited this article.

## Conflict of Interest

The authors declare that the research was conducted in the absence of any commercial or financial relationships that could be construed as a potential conflict of interest.

## Footnotes

^1^By 2019, the share of high school graduates among Roma had increased to a third and the percent of university graduates had risen to 3.5% of Roma aged up to 30, according to a recent, yet unpublished, study by Global Metrics Ltd. While this is remarkable progress, it continues to fall significantly short of national averages.

^2^Profs. Paul Gertler, Elise Huillery, and Joost de Laat.
